# Microscopic marine invertebrates are reservoirs for cryptic and diverse protists and fungi

**DOI:** 10.1186/s40168-022-01363-3

**Published:** 2022-09-30

**Authors:** Corey C. Holt, Vittorio Boscaro, Niels W. L. Van Steenkiste, Maria Herranz, Varsha Mathur, Nicholas A. T. Irwin, Gracy Buckholtz, Brian S. Leander, Patrick J. Keeling

**Affiliations:** 1 Department of Botany, University of British Columbia, Vancouver, Canada; 2 Hakai Institute, Heriot Bay, Canada; 3 Department of Zoology, University of British Columbia, Vancouver, Canada; 4 Department of Biology, University of Copenhagen, Copenhagen, Denmark

**Keywords:** Aquatic, Invertebrate, Microbiota, 18S, ASV, Symbiont, Host-associated

## Abstract

**Background:**

Microbial symbioses in marine invertebrates are commonplace. However, characterizations of invertebrate microbiomes are vastly outnumbered by those of vertebrates. Protists and fungi run the gamut of symbiosis, yet eukaryotic microbiome sequencing is rarely undertaken, with much of the focus on bacteria. To explore the importance of microscopic marine invertebrates as potential symbiont reservoirs, we used a phylogenetic-focused approach to analyze the host-associated eukaryotic microbiomes of 220 animal specimens spanning nine different animal phyla.

**Results:**

Our data expanded the traditional host range of several microbial taxa and identified numerous undescribed lineages. A lack of comparable reference sequences resulted in several cryptic clades within the Apicomplexa and Ciliophora and emphasized the potential for microbial invertebrates to harbor novel protistan and fungal diversity.

**Conclusions:**

Microscopic marine invertebrates, spanning a wide range of animal phyla, host various protist and fungal sequences and may therefore serve as a useful resource in the detection and characterization of undescribed symbioses.

Video Abstract

**Supplementary Information:**

The online version contains supplementary material available at 10.1186/s40168-022-01363-3.

## Background

The ubiquity of single-celled protists and fungi in various environments, alongside their ecological and metabolic diversity, has facilitated their capacity for niche exploitation. Some species support vast ecosystems as photosynthetic primary producers, while others utilize varying forms of heterotrophy, such as phagotrophs and parasites, which link ecological networks and trophic scales [1]. Indeed, all eukaryotic groups contain parasites that have evolved to exploit a host [2, 3]. The Apicomplexa, for example, form an exclusively symbiotic phylum (many being harmful parasites), characterized by the presence of a morphological structure called the apical complex, which aids host cell penetration and the initiation of infection [4]. However, relationships between protists, fungi, and metazoan hosts span the entire range of symbiosis. At the other end of the spectrum, photosynthetic dinoflagellates of the Symbiodiniaceae are well-known mutualists in coral [5], lignocellulose-degrading metamonads have facilitated niche expansion and the subsequent success of termites [6], while leucocoprineous fungi form a (typically) vertically transmitted association with fungus-growing attine ants [7].

Nevertheless, microbiome research has most commonly focused exclusively on bacterial communities, despite the understanding that microbial eukaryotes, along with archaea and viruses, are likely contributing to the pool of interactions that the term defines [3]. The lack of eukaryotic data in microbiome marker gene surveys is mostly due to methodological limitations rather than a genuine absence of protists. The co-amplification of host DNA when targeting symbiotic microbial eukaryotes can often dwarf non-metazoan reads and nullify attempts to fully characterize the eukaryome. However, various approaches have been developed to mitigate this problem, and sequence characterization of the eukaryotic microbiome is a possibility [8, 9].

The majority of microbiome studies focus on vertebrate hosts which ultimately represent a minute proportion of animal diversity [10, 11]. The Arthropoda alone makes up ~80% of all known animal species [11]. Microscopic marine invertebrates remain highly underrepresented in even bacterial microbiome literature [12]. As a consequence, eukaryotic microbiomes of these animals are almost completely unknown, and even for larger, commercially relevant marine invertebrates, the data are scarce [13].

Most invertebrate phyla include species smaller than 1–2 mm and belong to either planktonic or meiofaunal communities [14]; their abundance and diversity raise the possibility that microscopic marine invertebrates may interact with microbial eukaryotes in various ways. Many stable, symbiotic, microeukaryote-invertebrate associations are well documented [15–17], but protists can also be found inside/associated with these animals because they are consumed in their diet [18]. Therefore, the classification of microbial eukaryotes as true symbionts or components of a host-associated microbiome may be difficult with marker gene analysis alone.

Here, in an attempt to characterize protistan and fungal diversity in over 200 microscopic marine invertebrates, we rely on phylogenetic reconstruction to identify taxa that fall within typically host-associated clades, mitigating potential overemphasis and misidentification of microorganisms in the diet as symbionts. We expected that these minute animals could either be too small to host microbial eukaryotes, in which case we would not find sequence variants that could be reliably identified as symbiotic (i.e., fall within our target taxa), or simply understudied as viable hosts, which would result in the detection of a large proportion of unidentified lineages.

## Methods

### Specimen collection

**Fig. 1:**
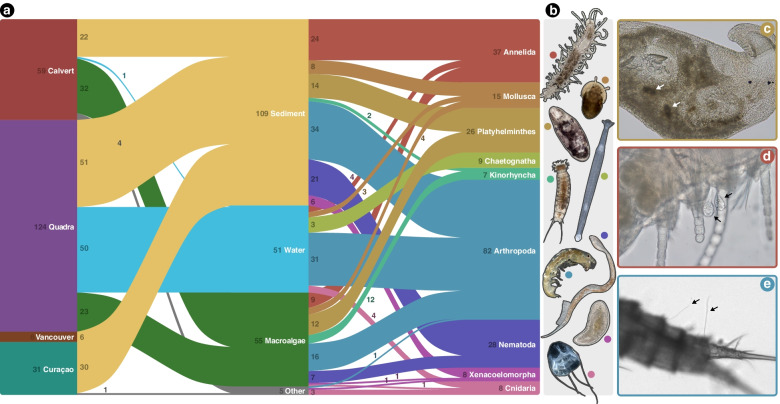
Overview of more than 200 specimens and visual evidence of symbiosis. **a** Sankey diagram showing the distribution of specimen locations, habitats, and phyla, with the number of specimens accompanying each factor. **b** Photos of representative specimens from each invertebrate phylum. **c** Unknown Apicomplexa shown in a Platyhelminthes specimen belonging to the family Koinocystididae. **d** Epibiont ciliates of the genus *Rhabdostyla* (black arrows) on an unknown species of a *Syllis* polychaete (Annelida). **e** Potential fungal structures on the dorsal side of a harpacticoid copepod. Image darkened to aid visibility

Microscopic invertebrate specimens were taken from a larger cohort of animals collected for bacterial microbiome analysis [12]. All specimens were isolated from one of three locations in British Columbia, Canada (Calvert Island, Quadra Island, and Vancouver) or Curaçao in the Dutch Caribbean, from July 2017 to January 2019. The majority of specimens were collected from either sediment, with a meiobenthic dredge (subtidal), or shovel (intertidal and subtidal); water column via horizontal and vertical plankton tows with a 64-μm mesh; or macroalgae, picked from rock pools. A small number of animals were sampled extemporaneously from other habitats, referred to as “other” in Fig. 1. The samples were taken back to the laboratory and stored at 4 °C. Animals were extracted from sediment and macroalgal samples with MgCl_2_ treatment [19] or the “bubble and blot” protocol [20], and specimens were isolated with Irwin Loops [21] under a dissecting microscope (Zeiss Stemi 508) within 24 h of arrival. All tools were sterilized with 10% bleach and 70% ethanol before use.

Prior to the preservation, specimens were transferred to droplets of sterile marine water, imaged on either a Zeiss Axioscope A1 or Leica DMIL microscope (British Columbia and Curaçao locations, respectively) with Axiocam 503 color or Sony a6000 cameras. Specimens were then assigned taxonomic groups following the World Register of Marine Species (WoRMS, https://www.marinespecies.org/index.php) and given a unique alphanumeric code. Recorded specimens were then washed in at least three successive transfers of sterile water and immediately frozen in 20 μL of sterile water at −20 °C until DNA extraction.

### DNA extraction and library preparation

DNA was extracted using the DNeasy PowerSoil Kit (QIAGEN Gmbh) according to the recommended protocol and quantified with the dsDNA HS Assay Kit (Life Technologies) and a Qubit Fluorometer. Amplicon libraries were generated using a nested PCR comprising an initial amplification with non-metazoan specific (UNon-Met-PCR) primers (18S-EUK581-F: 5’-GTGCCAGCAGCCGCG-3’, 18S-EUK1134-R: 5’-TTTAAGTTTCAGCCTTGCG-3’) [22]. UNon-Met-PCR primers not only significantly reduce metazoan reads but perform as well or better than common universal primers when amplifying the V4 region from a range of microeukaryotic taxa [23]. PCRs were performed in total volumes of 20 μL using Phusion High-Fidelity PCR Master Mix (New England BioLabs) and 2–4 μL of template DNA. Thermal cycler settings were as follows: initial denaturation, 98 °C (30 s); 35 cycles at 98 °C (10 s), 51 °C (30 s), and 72 °C (60 s); final extension, 72 °C (10 min). Amplicons were purified with the QIAquick PCR Purification Kit (QIAGEN) and quantified with a Qubit fluorometer before being sent to CGEB – Integrated Microbiome Resource for the subsequent PCR using “fusion primers” (Illumina adaptors + indices + specific regions) targeting the V4 region of the 18S rRNA gene (E572F: 5’-CYGCGGTAATTCCAGCTC-3’, E1009R: 5’-AYGGTATCTRATCRTCTTYG-3’) [24]. Fusion primer PCRs were performed in duplicate with 2 μL of the initial UNon-Met-PCR reaction, and one reaction using a 1/10th template dilution (as detailed on https://github.com/LangilleLab/microbiome_helper/wiki/Microbiome-Amplicon-Sequencing-Workflow). Duplicate libraries were then pooled, cleaned, and normalized with the Just-a-Plate 96-well Normalization Kit (Charm Biotech), and sequenced using 2 x 300 bp reads and Illumina Miseq v3 chemistry.

### Amplicon sequence variant (ASV) generation

Raw reads were initially trimmed with Cutadapt (v3.4) [25] to remove primers, before being processed in R [26] using the DADA2 package (v.1.14.1) [27]. Following the standard pipeline, trimmed reads were truncated based on quality profiles and filtered using the default parameters (maxN=0 and max EE=c(2,2)). Error rates were modeled on the first 100 million bases and “pseudo” pooling was implemented during sample inference to allow singletons, providing they appear in more than one library. Paired-end reads were then merged before chimera detection and taxonomic classification with the RDP Naïve Bayesian Classifier against the PR2 database (v.4.12.0) [28].

Preprocessing and filtering were done with the phyloseq package [29]. Libraries were removed if their relative abundance of metazoan reads was greater than 70%. Subsequently, all metazoan reads were removed from the remaining samples, as were non-eukaryotic sequences. Libraries with less than 1000 reads were discarded and any ASVs left with a read count of zero were also removed. To account for sequence variation across multiple copies of the 18S rRNA gene in individual protists, a phylogeny was reconstructed for all reads using IQTree (v.1.6.12) [30] and ASV sequences were subsequently grouped based on phyloseq’s tip_glom function (using Agnes hierarchical clustering and a tree height (h) of 0.05) [29]. The Sankey diagram, detailing specimen metadata of final library selection, was produced with the ggaluvial package in R [31].

### Phylogenetics

To reconstruct potential symbiont phylogenies, ASVs above a minimum relative abundance threshold of 0.1% in any one library were selected according to broad taxa assigned by the ASV pipeline (e.g., Apicomplexa, Ciliophora, Fungi, etc.). Unassigned sequences were also included if BLAST results showed similarities to the taxon in question. A selection of diverse, near full-length, reference sequences was added to structure each phylogeny including the top five BLAST hits (for each ASV) in the NCBI nt database (blastn, *e* value threshold of 1e−25). All sequences were trimmed to a maximum length of 2000 bp prior to aligning. The multiple sequence alignment was produced using the mafft EINSi iterative alignment algorithm [32] and masked with trimal to remove sites with gaps in more than 90% of sequences or with a similarity score of less than 0.001 [33]. A maximum-likelihood phylogeny was reconstructed with IQtree, using a GTR+F+R7 substitution model and 1000 ultrafast bootstraps [34]. The unaligned fasta files were trimmed to remove sequences that were irrelevant and/or represented minor strain variations from the same studies, and alignment, masking and phylogenies were repeated.

To visualize phylogenies, IQtree output trees were imported in R, rooted with the treeio package [35], and plotted using ggtree [36] and ggtreeExtra [37]. Branch lengths were removed to improve visualization of topologies, and bar charts displaying ASV prevalence measures in specimens were produced with ggplot2 [38] and later added using Adobe Illustrator.

## Results and discussion

### Specimen overview

The final dataset contains 220 isolated specimens: 56.4% of those were isolated from Quadra Island in British Columbia, Canada, and just under half of all specimens were isolated from the sediment (49.5%) (Fig. 1a). These animals span nine invertebrate phyla: Annelida, Mollusca, Platyhelminthes, Chaetognatha, Kinorhyncha, Arthropoda, Nematoda, Xenacoelomorpha, and Cnidaria (Fig. 1a, b). The most highly represented phylum was Arthropoda (*n*=82), followed by Annelida (*n*=37), and Nematoda (*n*=28) (Fig. 1a).

### Apicomplexa

**Fig. 2:**
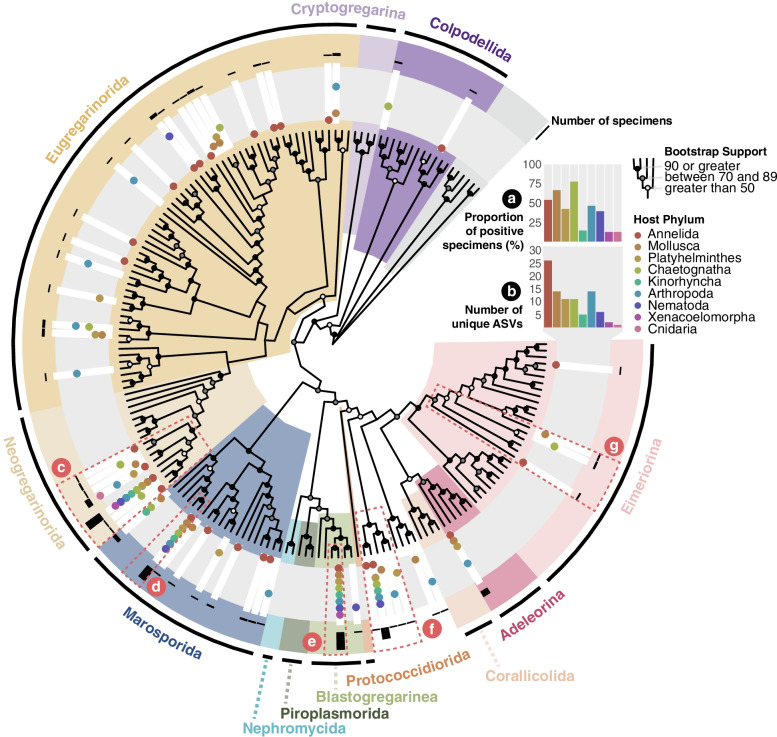
Apicomplexa prevalence and diversity. Maximum-likelihood phylogeny of all Apicomplexa ASVs, reference sequences, and BLAST hits using the GTR+F+R7 substitution model. Individual ASVs are indicated by white rectangles in the gray ring. Accompanying dots reflect the presence in each host phylum (colored accordingly). Black bars in the outer ring reflect the number of specimens associated with each ASV (on a log scale). Nodes are labeled to show UltraFast bootstrap support and taxonomic clades are annotated by color. **a** Percentage of individuals with at least one ASV in the tree. **b** Absolute number of distinct ASVs. **c**–**g** Highlighted lineages discussed in the text

A total of 52 ASVs were characterized as apicomplexans (Fig. 2, SFig. 1). The proportion of Apicomplexa-positive invertebrate specimens varied across phyla, ranging from just 12.5% of cnidarians (1/8) to 77.8% of chaetognaths (7/9) (Fig. 2a, STable 1). Notably, visual evidence of infection was observed in several platyhelminthes (which are typically less opaque than other animals in our dataset) (Fig. 1c); 42.3% of all platyhelminthes (11/26) contained at least one apicomplexan ASV (Fig. 2a, STable 1).

The Annelida contained the largest number of distinct apicomplexan sequences (Fig. 2b, STable 1); indeed, it is assumed that annelids are the ancestral hosts of gregarines (a subgroup of the Apicomplexa), before these parasites spread to other marine invertebrates [39]. Forty of the 52 ASVs were found in association with only a single host phylum, suggesting that many apicomplexans may have a high degree of host specificity. Although no single ASV was detected in all host phyla, six ASVs were found in four or more phyla. The majority of ASVs (*n*=19) were spread across known Eugregarinorida diversity, which is also true of other amplicon surveys [40].

Our data also provide evidence for wider host ranges of many known apicomplexan clades. For instance, one cluster of ASVs (spread across all nine host phyla) was found within the insect-infecting Neogregarinorida (Fig. 2c, Sfig.1), branching sister to a clade containing *Syncystis mirabilis* isolated from a “water scorpion” (*Nepa cincera*, Insecta), but also found in dragonflies, and *Quadruspinospora mexicana* from the Mexican lubber grasshopper (*Taeniopoda centurio*) [41]. Typically, the Neogregarinorida are known for infecting terrestrial hosts [42–45], and they are often found in amplicon surveys of soils and marine sediment [40]. Our phylogeny does include BLAST hits of environmental sequences isolated from soil (and sediment) within this cluster; therefore, we should not discount the idea that Neogregarinorida sequences found in our marine invertebrates could be derived from ingested cysts in terrestrial run-off. However, only 14 of the 46 occurrences of Neogregarinorida ASVs were from animals isolated from the sediment.

Gregarines, in general, are thought to be mostly monoxenous, meaning their life cycle involves just one host organism. Although two sequences of the Neogregarina were found in multiple host phyla (four and eight phyla respectively), most gregarine ASVs (20/27) were phylum-specific, with the remaining five ASVs found in just two host phyla (Fig. 2). Notably, two of these dixenous gregarines were detected in nematodes; six apicomplexan ASVs were detected in eleven individual nematodes in total (Fig. 2a, STable 1), despite no prior record of nematode-infecting Apicomplexa in the literature.

In the Marosporida, two ASVs found in various host phyla branched with a group of mollusc parasites as sisters to the Rhytidocystidae. ASV_713, found in a single kinorhynch, mollusc, and chaetognath, is the sister group of the remainder of this mollusc-infecting clade, whereas ASV_168 (one of our most widespread apicomplexan lineages and found in all host phyla except Cnidaria and Xenacoelomorpha) was identical to *Margolisiella islandica*, a heart-infecting parasite of the Islandic scallop *Chlamys islandica* [45] (Fig. 2d, SFig. 1). We also found several ASVs in the Rhytidocystidae—some of which were isolated from molluscs, platyhelminthes, and arthropods and therefore found outside of their typically associated hosts (annelids) [46].

The most abundant ASV is a sister lineage of the blastogregarine *Siedleckia* cf. *nematoides*, a parasite of the bristle worm (*Scoloplos armiger*), but only shares 87.32% sequence identity (Fig. 2e). This lineage was found in all phyla with the exception of Cnidaria. Another of the more abundant ASV branches as a novel clade within Coccidia (the subclass to which Corallicolida, Adeleorina, and Eimeriorina belong; Fig. 2). This position within the coccidia has low support (Fig. 2f, SFig. 1). Again, these cryptic sequences share low sequence similarity scores to GenBank accessions (< 85% to environmental sequences), and there appears to be no specific host and/or environmental trait consistent across all associated specimens in our dataset.

Finally, we detected lineages closely related to fish-infecting *Goussia* (Fig. 2g), supporting the hypothesis that small invertebrates may serve as paratenic hosts for some species. Three distinct sequences were found in two annelids, three chaetognaths, and two molluscs, respectively. The sequence found in annelids shares over 96% identity to *Goussia ameliae*, which was isolated from the pyloric caecum of landlocked alewives (*Alosa pseudoharengus*) and is not known to infect other hosts [47]. The chaetognath isolate is slightly more dissimilar (94.0% sequence identity) to the highest scoring reference sequence (*Goussia washuti* from wild bluegill, *Lepomis macrochirus*) [48] and likely represents an undescribed species. Finally, the molluscan sequence is closest to that of *Goussia pannonica* (99.2% sequence identity) from the blue bream (*Abramis* syn. *Ballerus sapa*) [49].

### Ciliophora

**Fig. 3:**
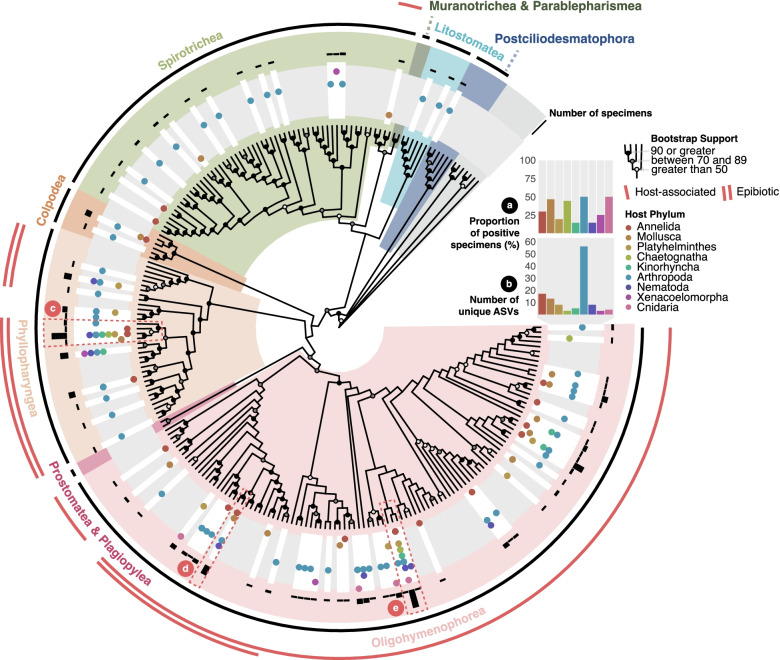
Environmental and host-associated ciliate lineages. Maximum-likelihood phylogeny of all Ciliophora ASVs, reference sequences and best BLAST hits using the GTR+F+R7 substitution model. Individual ASVs indicated by white rectangles in a gray ring. Accompanying dots reflect the presence in each host phylum (colored accordingly). Black bars in the outer ring reflect the number of specimens associated with each ASV (on a log scale). Nodes are labeled to show UltraFast bootstrap support and taxonomic clades are annotated by color. Outer red clade labels show host-associated taxa (single line) and epibiotic symbionts (double line). **a** Percentage of individuals with at least one ASV in the tree. **b** Absolute number of distinct ASVs. **c**–**e** Highlighted lineages discussed in the text

Contrary to apicomplexans, which are entirely host-restricted, most described ciliates are free-living. Consequently, the distribution of ciliate ASVs found in association with microscopic invertebrates does not match the taxonomic diversity and relative abundance predicted by environmental surveys of the group as a whole, but it does reflect what is known about lineages that are predominantly parasitic. We detected relatively few Spirotrichea (mostly oligotrichs and choreotrichs) and Litostomatea, which alone typically make up 70–90% of free-living ciliates in the marine environment [50]. ASVs belonging to these common ciliate groups are almost exclusively found in arthropods—animals at the largest end of the size range investigated here—and are most likely food. Indeed, nearly all ciliate ASVs were detected in arthropod specimens, 41 were detected in arthropods alone, and 50% (41/82) of all arthropod specimens contained at least one ciliate ASV (Fig. 3a, STable 1), resulting in the largest number of distinct ciliate ASVs compared to the other host phyla (Fig. 3b, STable 1). Half of all Cnidaria (4/8) also contained more than one ciliate ASV (although the total number of specimens analyzed was considerably lower). Conversely, ciliate ASVs were found in just 14.3% of Kinorhyncha and Nematoda (1/7 and 4/28, respectively) (Fig. 3a, STable 1).

The majority of ciliate ASVs in our dataset clearly belong to clades of known ecto- and endosymbionts, with a marked overrepresentation (compared to their relatively lower known diversity) of taxa from Suctoria and especially Apostomatia (epibiotic and parasitic subgroups of the Phyllopharyngea and Oligohymenophorea, respectively). Although most of these taxa are already known symbionts of marine invertebrates, they are generally documented in much larger specimens: adult echinoderms [51, 52], large cephalopods [53] and other molluscs [54], hydroids [55], and crustaceans [56]. Notably, associations between the suctorian genus, *Ephelota,* and small crustaceans (copepods) have also been reported [57, 58]. ASV_013, found in 18 host specimens across six phyla, formed a small cluster with other ASVs appearing as a sister group to species of *Ephelota* (Fig. 3c, SFig. 2). ASV_016 appears to be a member of the *Rhabdostyla* genus (Fig. 3d, SFig. 2), a well-known invertebrate epibiont and noted for their symbiotic relationship with annelids of the *Salvatoria* genus [59, 60]. We observed the same ciliate genus on a specimen of the *Syllis* polychaete (Fig. 1d). These epibionts are noted to sometimes result in the misidentification of some annelid species, given their morphological similarity to papillae [59].

Many of our ciliate ASVs were notably dissimilar from known reference sequences and often formed uninformative clusters. The most prevalent lineage (ASV_072) appeared in 24 animal specimens across seven of the nine phyla investigated. It branched in a weakly supported cluster with two, more spurious, ASVs and uncultured sequences from various marine environments, within the usually host-associated Oligohymenophorea (Fig. 3e, SFig. 2).

The detection of two *Colpoda*-like ASVs is unusual (Fig. 3, SFig. 2), given that the genus *Colpoda* is quintessentially terrestrial. Despite an old report (based on morphology) of a *Colpoda* commensal of the sea urchin (*Toxopneustes variegatus*) [61], and the existence of marine species within the class Colpodea [62, 63], these signals could also be soil-derived cysts ingested by the animals.

### Fungi

**Fig. 4:**
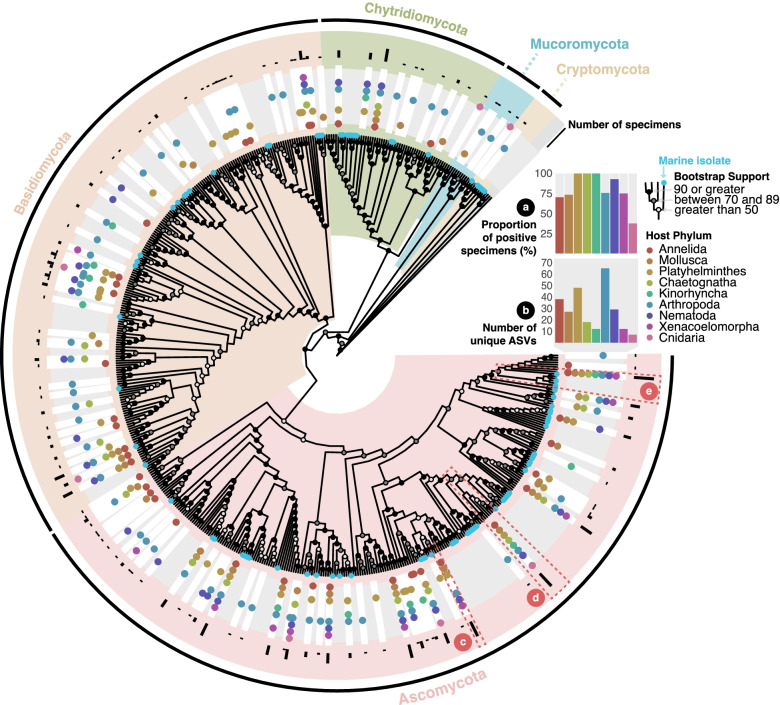
Evidence of fungal ASVs in marine invertebrates. Maximum-likelihood phylogeny of all Fungi ASVs, reference sequences and best BLAST hits using the GTR+F+R7 substitution model. Individual ASVs indicated by white rectangles in a gray ring. Accompanying dots reflect the presence in each host phylum (colored accordingly). Black bars in the outer ring reflect the number of specimens associated with each ASV (on a log scale). Nodes are labeled to show UltraFast bootstrap support and taxonomic clades are annotated by color. **a** Percentage of individuals with at least one ASV in the tree. **b** Absolute number of distinct ASVs. **c**–**e** Highlighted lineages discussed in the text

We detected a large diversity of fungal ASVs associated with marine invertebrates (Fig. 4, SFig. 3) and observed fungal-like structures emanating from some specimens (Fig. 1e). Many putatively marine fungi are assigned to species that are also found in terrestrial habitats—this is particularly true of the Ascomycota and Basidiomycota [64, 65] which make up the majority of species in our dataset (Fig. 4, SFig. 3). This may be indicative of terrestrial contamination, for instance, if marine invertebrates ingested spores, but fungal phylogenies often show putatively marine fungi nested within clades of typically terrestrial lineages [65]. This led to a hypothesis that most marine fungi diversified before animals transitioned to a terrestrial lifestyle [65], but it has also been proposed that many truly marine isolates recently evolved from terrestrial ancestors [64, 66]. Some fungi are capable of tolerating vastly different habitats [67], so may inhabit both marine and terrestrial environments. Our data does, however, support the idea that habitat can influence species localization [65, 68, 69]. Eighty-two of the 121 unique ASVs were from specimens localized to a single habitat; 49 were from sediment.

The Ascomycota and Basidiomycota represented 56 and 54 ASVs, respectively. By comparison, we found just ten ASVs belonging to the Chytridiomycota, which typically dominate other nearshore and sediment samples [64]. Fungal ASVs were found in the majority of specimens in all phyla except Cnidaria (where they were found in only 37.5% of specimens; Fig. 4a, STable 1). Furthermore, all Platyhelminthes, Chaetognatha, and Kinorhyncha contain at least one fungal ASV (Fig. 4a, STable 1). Despite this, there were relatively few unique fungal ASVs in both Chaetognatha and Kinorhyncha (Fig. 4b, STable 1). Of the total 121 unique fungal ASVs, 74 were found to be specific to just one host phylum, 25 of these host-phylum-specific sequences were found in arthropods and 19 in platyhelminthes. Although there is ample evidence of coevolution between fungal species and plant hosts [70], each host phylum-specific lineage in our dataset only ever occurred in one or two specimens. In contrast, two fungal sequences were found in more than 50 specimens and previous reports have shown how a single fungal species can engage with multiple ant genera [71]. ASV_117, found in six different host phyla, branches sister to a sequence from the Chaetothyriales (Fig. 4c, SFig. 3): often referred to as “black yeasts” and sometimes implicated as potentially pathogenic [72, 73].

ASV_019 is identical to several *Aspergillus* and *Penicillium* spp. (Fig. 4d, SFig. 3), which are often co-isolated from marine samples. *Aspergillus* spp. infect a wide range of vertebrate hosts, including cetaceans [74], and can produce metabolites detrimental to the photophysiological performance of the coral symbiont, *Symbiodinium* [75]. Both fungal genera have been isolated from diseased coral and sponges [76, 77]. Phylogenetic and microsatellite-based analyses have been unable to distinguish between aquatic and terrestrial strains of some species [78], but marine sequences are common [79] and ASV_019 was found in all host phyla except Xenacoelomorpha (Fig. 4d).

Our most common fungal sequence was found in all phyla except in the Cnidaria and appears to be related to *Cladosporium* spp., along with several uncultured sequences obtained from the marine environment (Fig. 4e, SFig. 3). Notably, *Cladosporium* produces an enzyme that digests phytoplankton-derived organic matter, and its abundance has been linked to diatoms in the ocean [80], which are likely ingested by our hosts. Some fungi, like the Cryptomycota (of which we detected one ASV), are indeed parasites of protists and other fungi [80].

### Other potential symbionts

#### Syndiniales

**Fig. 5:**
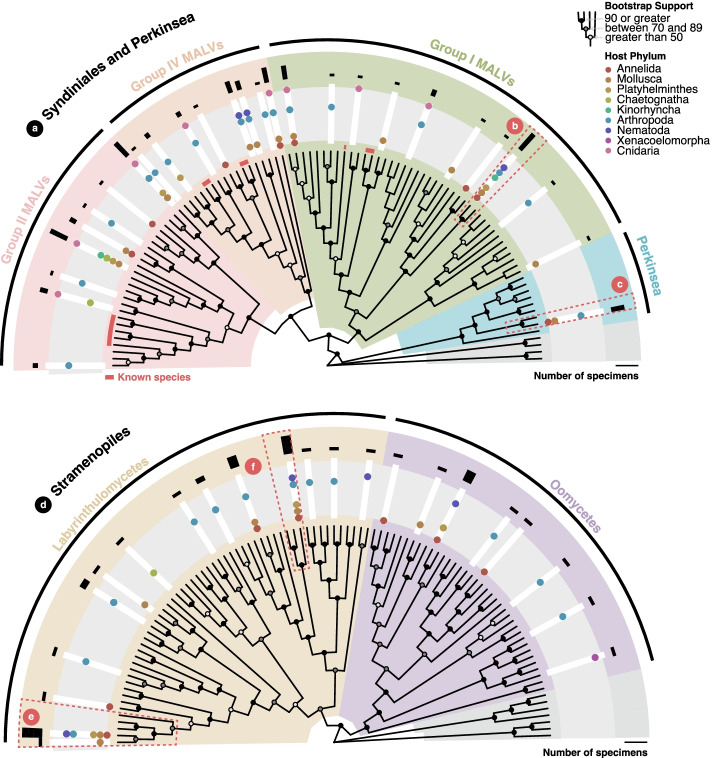
Other invertebrate symbiont taxa. Maximum-likelihood phylogeny of **a** marine alveolates (MALVs) and Perkinsea and **d** Stramenopiles ASVs, reference sequences and best BLAST hits using the GTR+F+R7 substitution model. Individual ASVs indicated by white rectangles in a gray ring. Accompanying dots reflect the presence in each host phylum (colored accordingly). Black bars in the outer ring reflect the number of specimens associated with each ASV (on a log scale). Nodes are labeled to show UltraFast bootstrap support and taxonomic clades are annotated by color. **b**, **c**, **e**, **f** Highlight lineages discussed in the text

Marine alveolates (MALVs), or Syndiniales, are thought to be exclusively parasitic lineages that form a paraphyletic group outside of the core dinoflagellate clade [81]. Despite often being the most dominant microbial eukaryote in environmental marker gene surveys [82, 83], the vast majority of Syndiniales are still uncultured, their hosts are unknown, and they are represented only by environmental sequences [84]. There are currently only five characterized species spread across three of the five recognized SSU rRNA clades (Groups I, II, and IV); Groups III and V are inferred only from environmental sequencing and are yet to be observed. We found Syndiniales in all invertebrate phyla except Xenacoelomorpha, with most ASVs being found in arthropods and molluscs (Fig. 5a, SFig. 4). This reflects our current understanding of these protists: Syndiniales are thought to be small flagellates that dominate seawater samples and would therefore be found in filter feeders like molluscs, and two of the five known Syndiniales genera typically infect arthropods.

Group II, in which the genus *Amoebophrya* is described, represents eight of our ASVs. *Amoebophrya* has been found in a wide range of dinoflagellate hosts and was recently estimated to represent eight different species [85]. Most of our Group II ASVs branched outside of the *Amoebophrya* clade. Of the further eight ASVs that fall within the Group IV Syndiniales, all but one were found in, but are not exclusive to, arthropods. Three of these sequences form orphan lineages that appear to have diverged prior to the clade containing both known Group IV genera: *Syndinium* (found in copepods and radiolarians [86]) and *Hematodinium* (found in crustaceans [87]). The most frequently detected Syndiniales sequence in our dataset (ASV_198) belongs to Group I (Fig. 5b, SFig. 4). However, it appears distinct from the two described genera within Group I: *Ichthyodinium* and *Euduboscquella* (syn. *Dubosquella*). *Ichthyodinium* spp. infect fish eggs [88] whereas *Euduboscquella* spp. are found in tintinnid ciliates [89]. ASV_198, found in the Annelida, Mollusca, Platyhelminthes, Kinorhyncha, Arthropoda, and Nematoda, branches sister to an environmental sequence from the Northwest Pacific Ocean. We did, however, find ASVs that shared much more recent ancestors with both Group I type species. Furthermore, most of our Syndiniales likely fall in Group I, which is also noted to be the dominant group in other zooplanktonic hosts [90].

#### Perkinsea

Perkinsea are alveolate parasites that can cause mass mortality events in fish, molluscs, and amphibians [91]. We detected a single perkinsid ASV in seven microscopic invertebrate specimens (two annelids, two molluscs, and three arthropods) (Fig. 5c, SFig. 4). Notably, this sequence is nearly 96% identical to that of *Perkinsus qugwadi*, a species that has caused sporadic mass mortality events in Yesso scallop (*Patinopecten yessoensis*) stocks in British Columbia [92]. Given that *P. qugwadi* shares ~96% sequence identity with some other *Perkinsus* species, it is likely we have detected a novel but related species. Although, notably, all of our associated specimens were isolated from the same location as previous *P. qugwadi* outbreaks (Quadra Island) [92, 93].

#### Stramenopiles

We generally found lower proportions of specimens with potentially host-associated stramenopile ASVs—ranging from zero kinorhynchs and cnidarians to 26.7% (4/15) of molluscs (Fig. 5d, SFig. 4). The most prevalent sequence was found in just seven individual hosts and appears to be related to the *Labyrinthula* genus (Fig. 5e, SFig. 4), a well-known pathogen of various seagrass species and also noted for its association with other algae and phytoplankton [94]. These seven specimens were not however isolated for macroalgae, but rather sediment.

Several studies describe pathologies caused by thraustochytrids in large molluscs [95], and it has been suggested that a specific pathological association may exist. However, most of our sequences within the Thraustochytrida were isolated from host phyla other than Mollusca; ASV_430 was found in one molluscan specimen (Solenogastres), but not exclusively (Fig. 5f, SFig. 4). Sequences from both of these Labyrinthulomycetes orders (Labyrinthulida, to which the abovementioned *Labyrinthula* belongs, and Thraustochytrida) could represent saprotrophic organisms; however, several invertebrate associations exist [96, 97]. The same can be said of the oomycetes, which are notable for their wide host and geographic range [98–100].

## Conclusions

Our sampling uncovers a role for microscopic invertebrates in the ecology of microbial eukaryotes. We detected a wide range of diverse organisms, often expanding the host range of previously characterized microbes, and several clades that we could not identify using presently archived reference sequences. Thus, our data support the hypothesis that, despite their size, microscopic marine invertebrates still harbor protist and fungal symbionts—many of which are currently uncharacterized.

It should be noted that short regions of the 18S SSU gene alone limit our ability to distinguish unique species; even distinct protistan species can have almost identical 18S genes [101]. Utilizing the full length of the 18S gene sequence would be the next step in improving the taxonomic resolution of our potential symbionts [102].

Although we acknowledge the potential for terrestrial run-off, these works support the notion that protists and fungi should be included in analyses of invertebrate microbiomes, and highlight host taxa that could warrant further exploration.

## Supplementary Information

**Additional file 1: Supplementary Table 1.** Number of Apicomplexa, Ciliophora and Fungi ASVs in each host phylum. n.pos = number of specimens positive for microeukaryote in question, spec.total = total number of specimens, prop.pos = proportion of positive specimens (%), n.uniq.ASVs = number of unique ASVs per host phylum.

**Additional file 2: Supplementary Figure 1.** Apicomplexa phylogeny with sequence labels and UltraFast bootstrap support values. Maximum-likelihood phylogeny of all Apicomplexa ASVs, reference sequences, and BLAST hits using the GTR+F+R7 substitution model. Individual ASVs indicated by bold tip labels and white rectangles in grey ring. Accompanying dots reflect presence in each host phylum (coloured accordingly).

**Additional file 3: Supplementary Figure 2.** Ciliophora phylogeny with sequence labels and UltraFast bootstrap support values. Maximum-likelihood phylogeny of all Ciliophora ASVs, reference sequences, and BLAST hits using the GTR+F+R7 substitution model. Individual ASVs indicated by bold tip labels and white rectangles in grey ring. Accompanying dots reflect presence in each host phylum (coloured accordingly).

**Additional file 4: Supplementary Figure 3.** Fungi phylogeny with sequence labels and UltraFast bootstrap support values. Maximum-likelihood phylogeny of all Fungi ASVs, reference sequences, and BLAST hits using the GTR+F+R7 substitution model. Individual ASVs indicated by bold tip labels and white rectangles in grey ring. Accompanying dots reflect presence in each host phylum (coloured accordingly).

**Additional file 5: Supplementary Figure 4.** Marine alveolate (top) and Stramenopiles (bottom) phylogenies with sequence labels and UltraFast bootstrap support values. Maximum-likelihood phylogeny of all MALV and host-associated Stramenopiles ASVs, reference sequences, and BLAST hits using the GTR+F+R7 substitution model. Individual ASVs indicated by bold tip labels and white rectangles in grey ring. Accompanying dots reflect presence in each host phylum (coloured accordingly).

## Data Availability

All sequence data are deposited in the NCBI Short Read Archive under the BioProject accession number PRJNA746569.
