# Supplementary figures and images for: Microscopic marine invertebrates are reservoirs for cryptic and diverse protists and fungi
Source: Microbiome. 2022 Sep 30;10:161. doi: 10.1186/s40168-022-01363-3 (PMC9523941; doi:10.1186/s40168-022-01363-3)

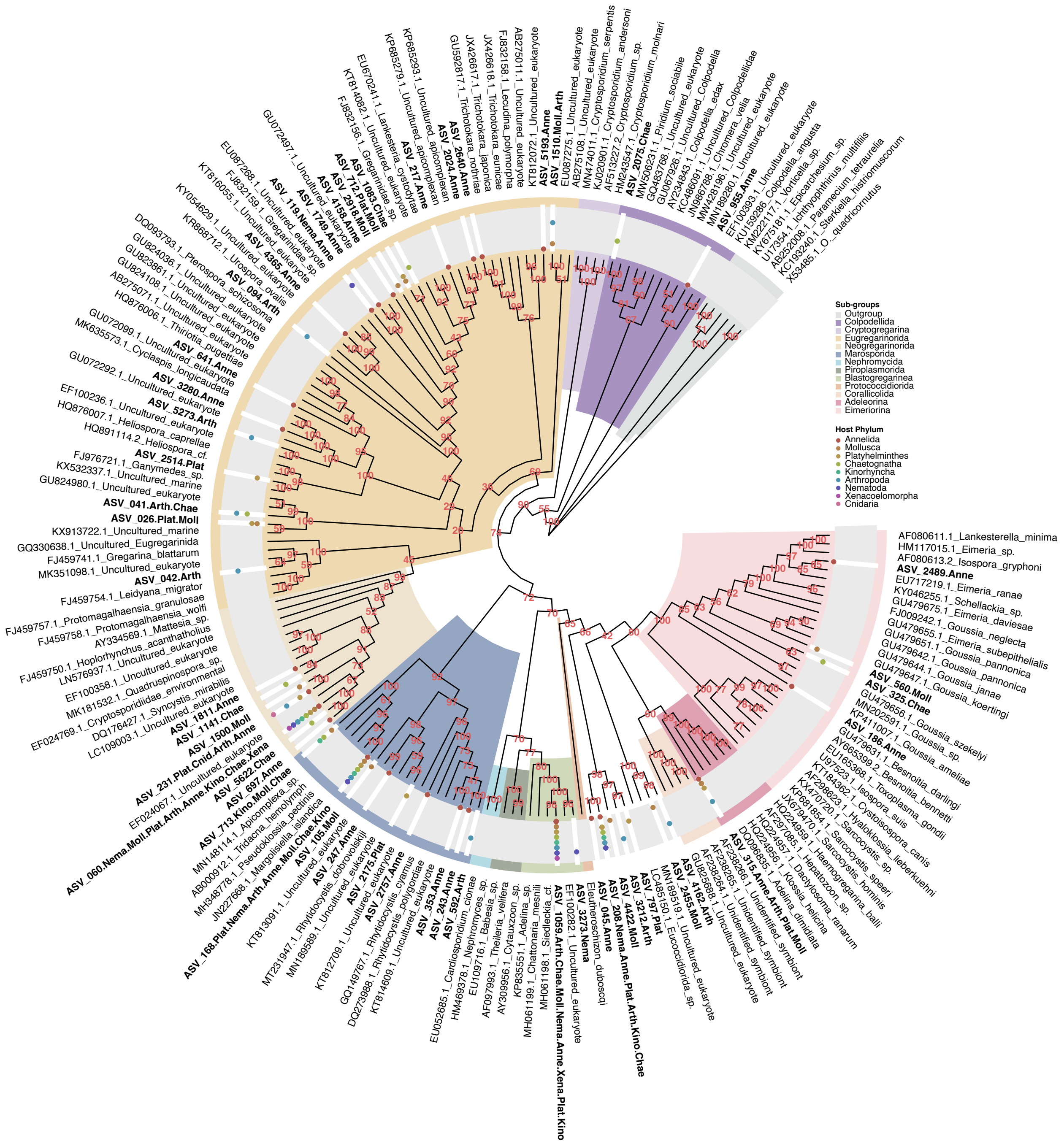

Supplement: Supplementary file 3 — Additional file 2: Supplementary Figure 1. Apicomplexa phylogeny with sequence labels and UltraFast bootstrap support values. Maximum-likelihood phylogeny of all Apicomplexa ASVs, reference sequences, and BLAST hits using the GTR+F+R7 substitution model. Individual ASVs indicated by bold tip labels and white rectangles in grey ring. Accompanying dots reflect presence in each host phylum (coloured accordingly). [file 40168_2022_1363_MOESM2_ESM.pdf]

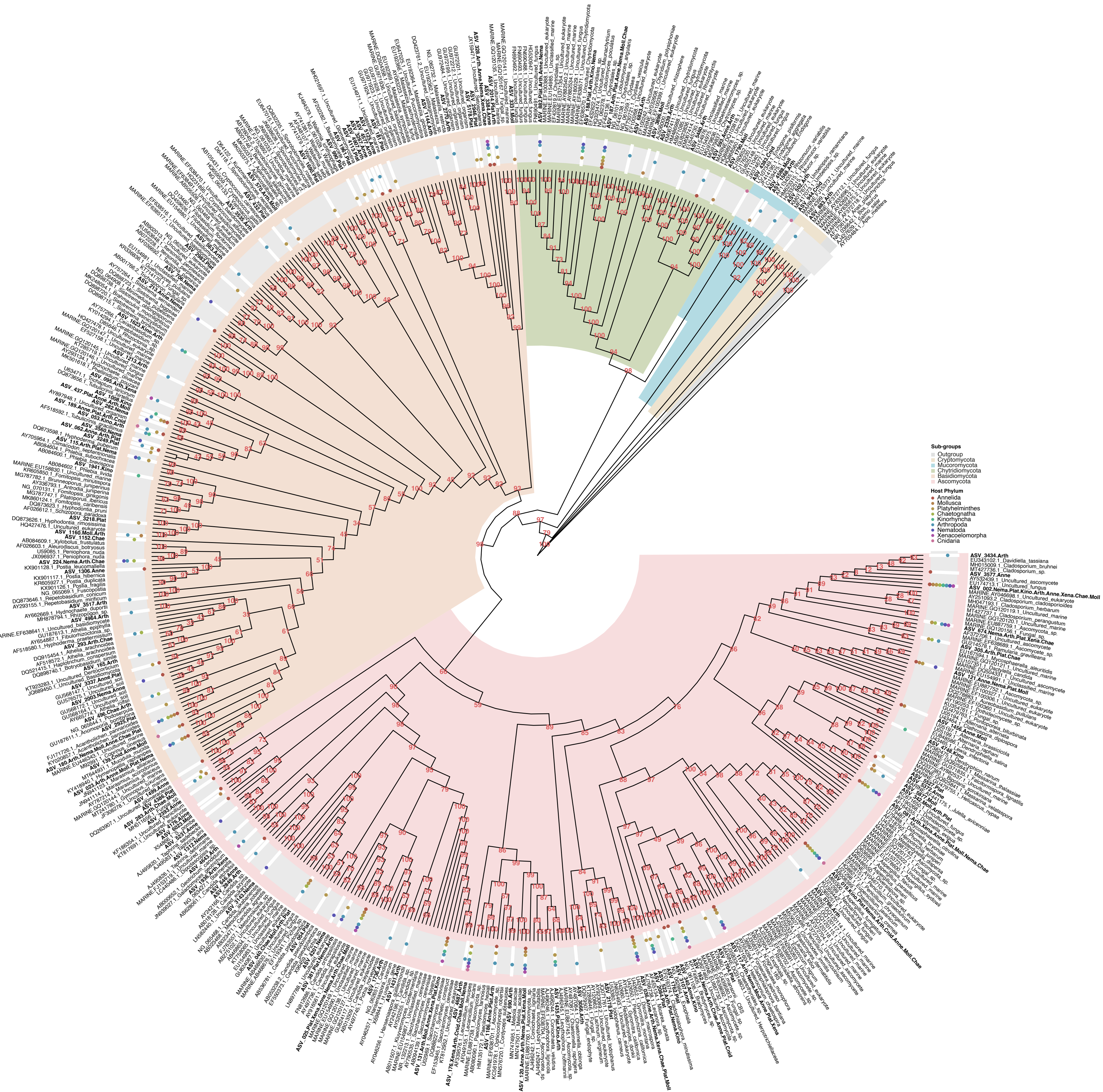

Supplement: Supplementary file 4 — Additional file 3: Supplementary Figure 2. Ciliophora phylogeny with sequence labels and UltraFast bootstrap support values. Maximum-likelihood phylogeny of all Ciliophora ASVs, reference sequences, and BLAST hits using the GTR+F+R7 substitution model. Individual ASVs indicated by bold tip labels and white rectangles in grey ring. Accompanying dots reflect presence in each host phylum (coloured accordingly). [file 40168_2022_1363_MOESM3_ESM.pdf]

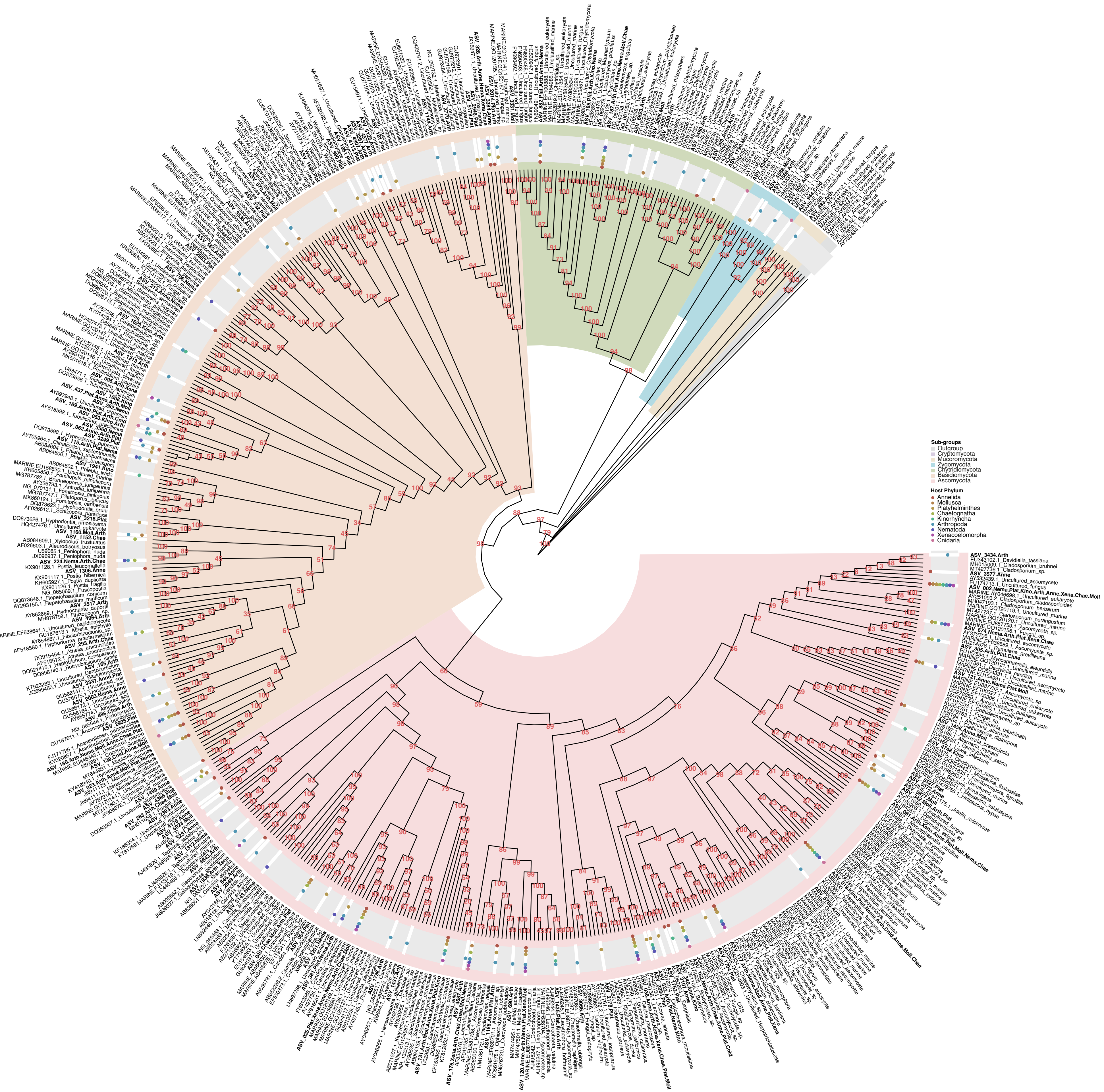

Supplement: Supplementary file 5 — Additional file 4: Supplementary Figure 3. Fungi phylogeny with sequence labels and UltraFast bootstrap support values. Maximum-likelihood phylogeny of all Fungi ASVs, reference sequences, and BLAST hits using the GTR+F+R7 substitution model. Individual ASVs indicated by bold tip labels and white rectangles in grey ring. Accompanying dots reflect presence in each host phylum (coloured accordingly). [file 40168_2022_1363_MOESM4_ESM.pdf]

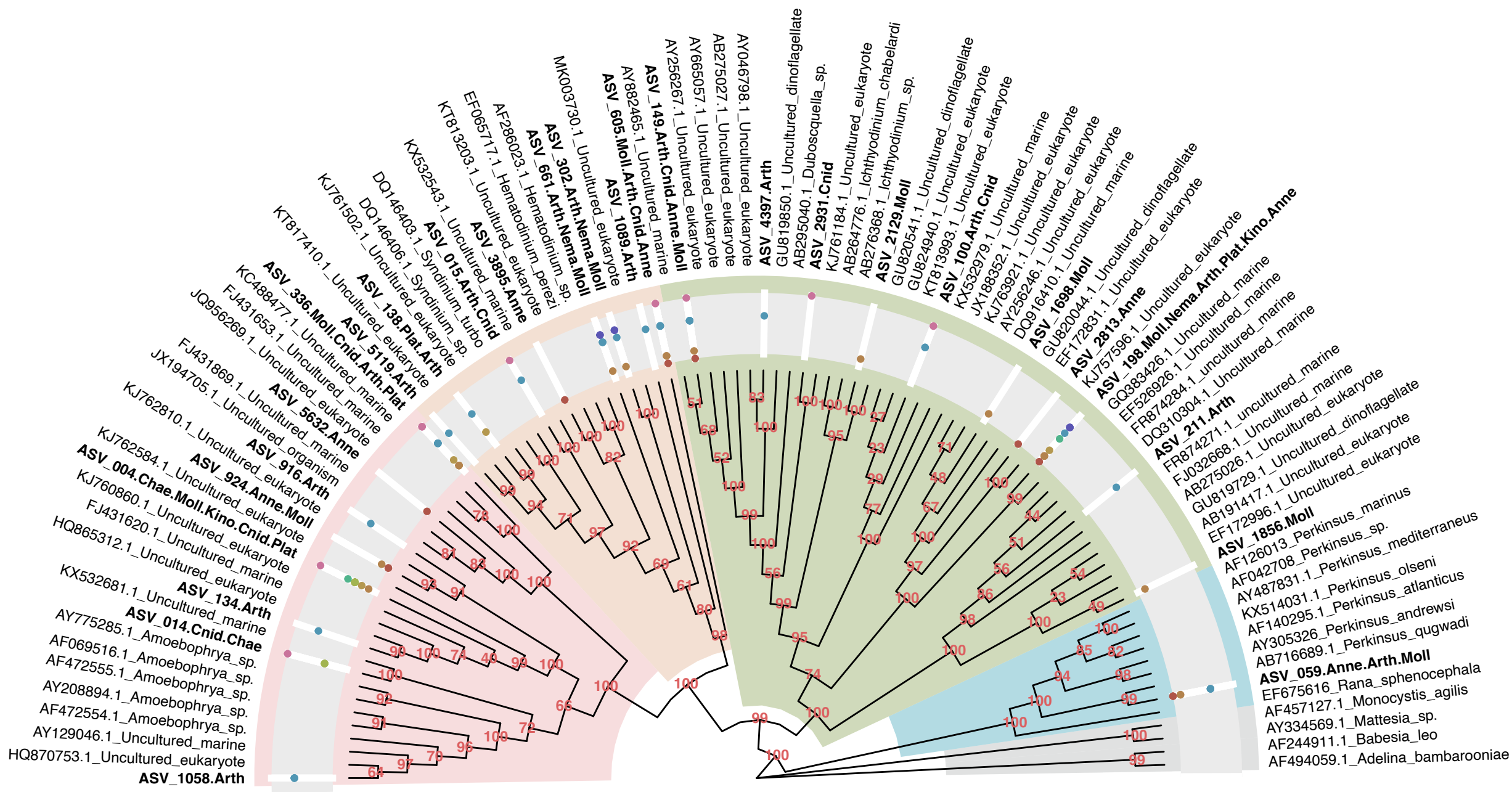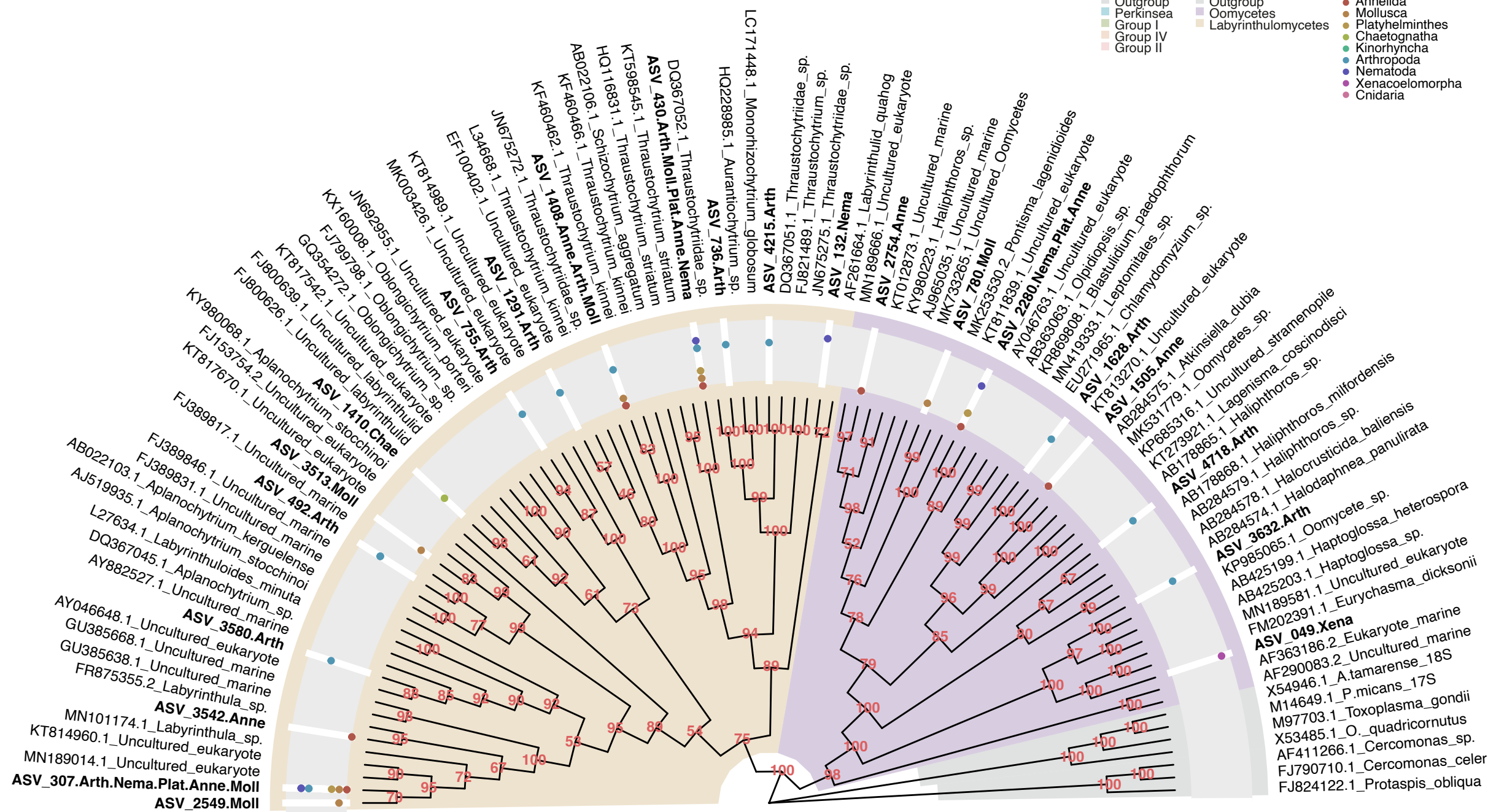

Supplement: Supplementary file 6 — Additional file 5: Supplementary Figure 4. Marine alveolate (top) and Stramenopiles (bottom) phylogenies with sequence labels and UltraFast bootstrap support values. Maximum-likelihood phylogeny of all MALV and host-associated Stramenopiles ASVs, reference sequences, and BLAST hits using the GTR+F+R7 substitution model. Individual ASVs indicated by bold tip labels and white rectangles in grey ring. Accompanying dots reflect presence in each host phylum (coloured accordingly). [file 40168_2022_1363_MOESM5_ESM.pdf]
